# Phylogenetic Analysis of Glycerol 3-Phosphate Acyltransferases in Opisthokonts Reveals Unexpected Ancestral Complexity and Novel Modern Biosynthetic Components

**DOI:** 10.1371/journal.pone.0110684

**Published:** 2014-10-23

**Authors:** Heather C. Smart, Fred D. Mast, Maxwell F. J. Chilije, Marjan Tavassoli, Joel B. Dacks, Vanina Zaremberg

**Affiliations:** 1 Department of Biological Sciences, University of Calgary, Calgary, Alberta, Canada; 2 Department of Cell Biology, University of Alberta, Edmonton, Alberta, Canada; Simon Fraser University, Canada

## Abstract

Glycerolipid synthesis represents a central metabolic process of all forms of life. In the last decade multiple genes coding for enzymes responsible for the first step of the pathway, catalyzed by glycerol 3-phosphate acyltransferase (GPAT), have been described, and characterized primarily in model organisms like *Saccharomyces cerevisiae* and mice. Notoriously, the fungal enzymes share low sequence identity with their known animal counterparts, and the nature of their homology is unclear. Furthermore, two mitochondrial GPAT isoforms have been described in animal cells, while no such enzymes have been identified in Fungi. In order to determine if the yeast and mammalian GPATs are representative of the set of enzymes present in their respective groups, and to test the hypothesis that metazoan orthologues are indeed absent from the fungal clade, a comparative genomic and phylogenetic analysis was performed including organisms spanning the breadth of the Opisthokonta supergroup. Surprisingly, our study unveiled the presence of ‘fungal’ orthologs in the basal taxa of the holozoa and ‘animal’ orthologues in the basal holomycetes. This includes a novel clade of fungal homologues, with putative peroxisomal targeting signals, of the mitochondrial/peroxisomal acyltransferases in Metazoa, thus potentially representing an undescribed metabolic capacity in the Fungi. The overall distribution of GPAT homologues is suggestive of high relative complexity in the ancestors of the opisthokont clade, followed by loss and sculpting of the complement in the descendent lineages. Divergence from a general versatile metabolic model, present in ancestrally deduced GPAT complements, points to distinctive contributions of each GPAT isoform to lipid metabolism and homeostasis in contemporary organisms like humans and their fungal pathogens.

## Introduction

**Figure 1:**
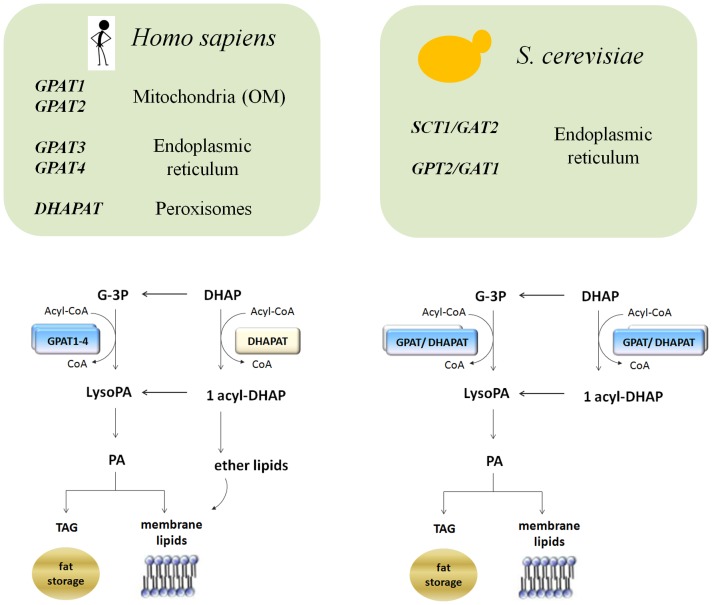
Glycerolipid biosynthesis in eukaryotes. Cartoons highlighting differences and similarities between the glycerolipid biosynthetic pathways in humans and yeast *Saccharomyces cerevisiae*.

Glycerolipids are the most abundant lipid class in eukaryotes and, although they were first regarded as static structural membrane components and energy storage molecules, they are now also recognized as dynamic players in cellular processes regulating a wide range of signaling pathways and organelle specific functions [1], [2], [3]. The *de novo* pathway for the synthesis of glycerolipids (Kennedy pathway) initially involves two consecutive acylation events where acyl groups from acyl-CoA are transferred to the *sn-1* and *sn-2* positions of glycerol 3-phosphate (G3P) to form phosphatidic acid (PA). The first of these acylation events is specific for the *sn-1* position of G3P and is catalyzed by glycerol-3-phosphate acyltransferase (GPAT) (Figure 1) to produce lysophosphatidic acid (LysoPA). The GPAT step is considered the committed and rate-limiting step of the pathway, representing an important control point in the flow of fatty acids and glycolytic metabolites into both functional and structural membrane components, or fat storage in the form of triacylglycerol [4], [5], [6]. It is important to highlight that the “acyl-CoA” substrate in the GPAT reaction represents many possible acyl chains varying in length and degree of unsaturation. Favoring the incorporation of a specific pool of acyl-CoAs into PA will depend on fatty acid availability and substrate preference of the acyltransferases involved. If, in addition to substrate preference, we consider that different subcellular compartments may rely on specific GPAT isoforms to provide particular PA pools for optimum organelle function, it is therefore not surprising to find that acyltransferases are highly redundant within eukaryotes.

For several decades after the identification of bacterial and mitochondrial GPATs [7], [8], eukaryotic microsomal GPATs remained elusive, and just recently the discovery of new genes encoding these enzymes has flourished [9], [10], [11]. Major contributions to the identification of the genes involved in the *de novo* glycerolipid biosynthetic pathways came from studies in model organisms like mice and yeast. Four mammalian GPAT isoforms encoded by independent genes have been identified to date, two of them mainly localized to mitochondria (GPAT1 and GPAT2) and the other two (GPAT3 and GPAT4) associated with the endoplasmic reticulum (ER) [10], [12]. Animals also contain an additional acyltransferase localized to peroxisomes which catalyzes a similar reaction, but uses dihydroxy-acetone phosphate (DHAP) instead of G3P, therefore called dihydroxy-acetone phosphate acyltransferase (DHAPAT) [13]. The product of this reaction, 1-acyl-DHAP, is normally the precursor for the synthesis of ether lipids in peroxisomes [14]. In addition 1-acyl-DHAP can also be converted to lysoPA by a reductive step feeding the Kennedy pathway for synthesis of PA [15]. In the yeast *Saccharomyces cerevisiae*, two unique genes, *SCT1/GAT2* and *GPT2/GAT1*, code for two GPATs, both of which are integral membrane proteins located in the endoplasmic reticulum (ER) [11], [16]. Unlike mammalian GPATs, the yeast GPAT proteins are dual specificity acyltransferases, catalyzing the addition of acyl-CoA to both G3P and DHAP, although their preference for DHAP differs [11]. The purpose of the DHAP shunt in yeast is not clear. *S. cerevisiae* does not possess a peroxisomal DHAPAT and this kind of enzyme has not been identified in any other fungus to date. Furthermore, analysis of the yeast lipidome has failed to identify the presence of ether lipids in this organism [17].

The GPAT/DHAPAT proteins of both yeast and humans belong to the lysophospholipid acyltransferase (LPLAT) superfamily which contain a signature acyltransferase domain (pfam01553) consisting of four distinct motifs (motif I, II, III and IV) arranged sequentially [18], [19]. A striking difference between the GPAT proteins identified in yeast and those in mammals is the distance between motifs II and III, with 109 versus 25 residues separating these motifs in yeast and mammalian GPATs respectively [20]. The yeast GPATs share high sequence similarity but differentially contribute to triacylglycerol and phospholipid biosynthetic pathways [21]. In addition Sct1 appears to have a preference for the incorporation of palmitate while Gpt2 is also able to utilize oleate [6], [22]. Unique expression patterns have been described for each of the animal acyltransferases in adult rodents [10], [12] and during distinct stages of embryonic development in *Xenopus laevis* [23]. Animal mitochondrial GPATs (GPAT1 and GPAT2) and the microsomal GPATs (GPAT3 and GPAT4) all contribute to TAG synthesis, but a differential involvement in phospholipid synthesis by distinct isoforms has also been postulated [5], [24], [25], [26]. Furthermore, recent evidence supports a critical role for the PA pool that results from GPAT activity in the regulation of signaling pathways that control cell proliferation [27], [28].

Therefore, redundancy and differential partitioning of lipids metabolized by unique GPATs is observed from yeast to man, but intriguingly, the fungal enzymes share low sequence identity (∼11%) with their known animal counterparts. Since these enzymes are key metabolic players in eukaryotic cells, their evolutionary history is an important component to understanding the cellular evolution of membrane biogenesis, trafficking, signalling and to linking organellar and metabolic evolution in this set of eukaryotic taxa. In this work, we aimed to unravel the evolutionary history of these proteins in opisthokonts, which includes fungi and metazoans, in order to: determine how representative the *S. cerevisiae* GPAT complement is of other fungal taxa; how representative the mammalian complement is of animal taxa; and critically assess how the sets of enzymes correspond to one another.

## Materials and Methods

### Comparative Genomic Survey

**Figure 2:**
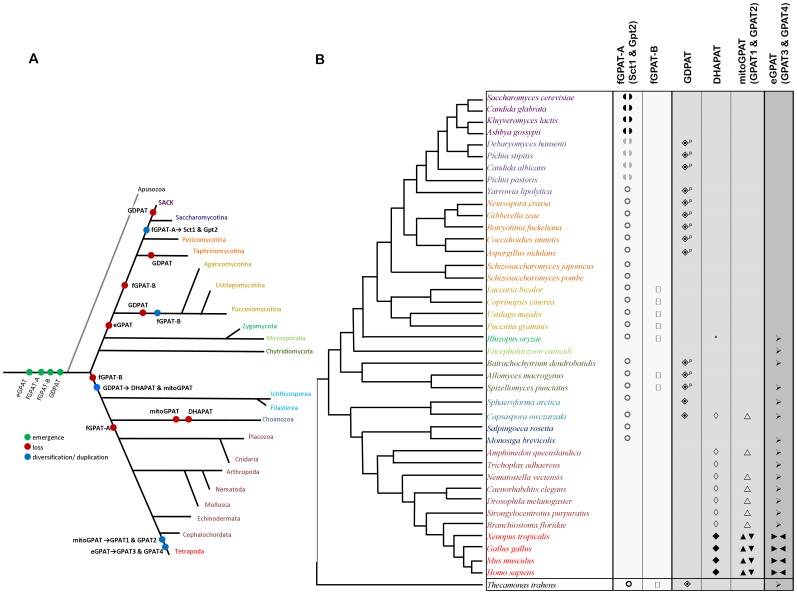
Opisthokont relationships and GPAT evolution. **A**) Tree illustrating the relative evolutionary history for the eukaryotic emergence, loss and diversification/duplication of GPATs in opisthokonts, in lineages with genome sequences available in the fungi and metazoans with additional taxa from the base of the Opisthokonta supergroup. The apusozoan, *T. trahens*, was used as the outgroup. The group labeled “SACK” belongs to Saccharomycotina and involves *S. cerevisiae, A. gossypii, C.glabrata*, and *K. lactis* To note the Pezizomycotina and Taphrinomycotina form a paraphyletic group of ‘non- Saccharomycotina ascomycota’ which we found to be a useful grouping when considering the evolution of GPATs and are thus colour coded identically. Similarly, all non-tetrapoda metazoans are coded in the same colour. In order to avoid false negative or false positives in our evolutionary deductions, we do not infer any events on lineages where only a single genome was examined. **B**) Results from the comparative genomic survey and phylogenetic analysis. Individual species from the survey are color coded as in A) and grouped according to established taxonomic classification. The dendrogram is schematic of relationships only and the branch length is not representative of evolutionary distance. Symbols indicate the presence of *at least* one isoform of a given protein as verified by BLAST, Reciprocal BLAST, hidden Markov model searches, and phylogenetic data as follow: orthologs of Sct1/Gat2(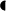), Gpt2/Gat1(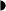), as well as new fungal fGPAT-A (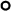) or fGPAT-B (**□**) and orthologs of human mitochondrial GPATs (mitoGPATs) GPAT1(▴), GPAT2(▾), putative mitoGPATs (▵), GDPAT (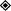), DHAPATs (⧫) and putative DHAPATs (pDHAPAT ◊), and orthologs of human microsomal GPATs (eGPATs) GPAT3(▸) and GPAT4(◂) and putative eGPATs (→). Putative orthologs of Sct1 and Gpt2 are shown in grey while strongly supported orthologs are shown in solid black. Blank cells indicate no hits. (^p^) denotes GDPATs with PTS1 prediction. Designation in a particular orthologous group denotes a prediction of substrate specificity, based on assumed retention between orthologues, and provides hypotheses for future functional testing. (*) The Rhizopus acyltransferase gene groups with moderate support with the DHAPAT genes, but this placement may well be due to its highly divergent sequence. Protein sequences used to initiate the searches: yeast Sct1 (NP_009542.1), yeast Gpt2 (NP_012993.1), human GPAT1 (NP_065969.3), human GPAT2 (NP_997211.2), human DHAPAT (NP_055051.1), human GPAT3 (NP_116106.2), human GPAT4 (NP_848934.1). *Note*: Putative fungal GDPATs display a peroxisomal target sequence (PTS1) and were hits in a search initiated with mitochondrial human GPAT1.

Organisms studied: A variety of organisms from both Metazoa and Fungi, with readily available protein reference sequence databases, were used to probe for both human-like and yeast-like GPATs. The organisms used are listed in Table S1, and were chosen to represent a broad range of organisms from both the fungal and metazoan group, while maintaining simplicity, and focusing on close relatives of yeast (Ascomycota) and humans (Metazoa). In order to bridge the gap between the model systems within the fungi and animals, organisms that diverge paraphyletically from the base of the metazoan and fungal lineages were included (Figure 2). For comparison, the eukaryote lineage of apusozoa (*Thecamonas trahens*) was used as the outgroup. Although organisms with fully sequenced genomes were chosen wherever possible, some organisms in key lineages with unpublished or incomplete genomes were included, specifically: *Batrachochytrium dendrobatidis, Spizellomyces punctatus, Allomyces macrogynus, Salpingoeca rosetta, Sphaeroforma arctica, Capsaspora owczarzaki* and *Thecamonas trahens*.

Putative yeast GPAT homologues were identified across the organisms listed in Table S1 through a process of iterative searches using Basic Local Alignment Search Tool (BLAST 2.2.22) [29] for proteins (BLASTp) and used to build a profile hidden Markov model (HMM) using HMMer 2.3.3 [30]. First, publicly hosted genomes at the NCBI using BLASTp were surveyed using the yeast GPATs as queries with a cutoff expect (E) value for positive candidate homologues set at 0.05. Genomes not hosted by the NCBI were queried locally using the BLASTp program. In order to validate putative candidates, putative homologue sequences were used as BLASTp queries into the *S. cerevisiae* genome and considered validated if they returned one of the functionally characterized GPAT protein sequence as the best scoring retrieved sequence (Reciprocal BLAST). The same process was repeated with the human mitochondrial and microsomal GPATs, using reciprocal blast into *Homo sapiens* to validate putative homologues.

Validated sequences were aligned using MUSCLE 3.6 (Multiple sequence alignment (MSA) tool) [31] and used to build a Hidden Markov Model (HMM) using a maximum likelihood (ML) architecture construction algorithm [30]. This model was then used to search the locally hosted genomes listed in Table S1 for putative homologues. Newly identified sequences below the 0.05 E value cutoff were validated by Reciprocal BLAST and added to the HMM iteratively until all genomes had been searched and the list of candidate homologues was exhausted. Due to the observed similarity between sequences, the following GPATs were grouped together into HMM matrices to improve searching criteria; yeast Sct1 & Gpt2, human GPAT1 & GPAT2, and human GPAT3 & GPAT4.

### Alignment and Phylogenetic Analysis

Phylogenetic analysis was performed on both the validated homologues of yeast Sct1 & Gpt2 (fungal GPATs) and human GPAT1 & GPAT2 (mitochondrial GPATs). Alignments were created using MUSCLE and then visually inspected and masked using MESQUITE version 2.75 [32] leaving only regions of unambiguous homology. All alignments are available from the authors upon request.

The masked alignment was analyzed using ProtTest version 1.3 [33] to identify the optimal model of sequence evolution, incorporating amino acid transition matrices, invariable sites and rate-among-site corrections as appropriate. RaxML 7.0.4 [34] was used to create 100 non parametric bootstrap trees using the identified model. A single bootstrap consensus tree was then created using Consense within the Phylip 3.69 package [35]. The same parameters were used with PhyML [36] to generate a ML tree. MrBayes version 3.2 [37] was used to create an optimal tree topology and posterior probability values. Analyses were run for 1,000,000 Markov chain Monte Carlo generations, and the burn-in values were obtained by ensuring that two independent runs had converged with a split frequency of 0.1 and by removing all trees prior to a graphically determined plateau of log likelihood values. The three generated trees, using all methods (MrBayes, PhyML and RaxML) were analyzed, and highly divergent sequences (i.e. as assessed by branch-length) that were not central to the question being addressed were removed (data not shown). The trees were then re-generated using the same strategy.

Further validation of identified GPAT homologs was performed by observing the amino acid sequence which aligned with the catalytic motifs of known GPATs. The un-masked MSA, of the sequences previously used to generate each phylogenetic tree, was visually inspected for the region containing the catalytic motifs, and the sequence of the alignment at the same position for each identified protein was recorded. Also, the distance between motifs (DBM) was catalogued by computing the number of amino acid residues in each full protein sequence between motifs I & II, II & III and III & IV.

All accession numbers can be found in Tables S3, S4 and S5.

## Results

### Searching for Sct1/Gpt2 identifies two undescribed deep clades of fungal GPATs

Our first goal was to organize all available genomic data into a structure that could aid in the assessment of the evolutionary relatedness among GPATs in animal and fungal species and also to generate clusters of related sequences in order to identify unique protein features within each group. For this purpose a comparative genomic analysis restricted to the super group Opisthokonta was initiated. Although opisthokonts represent only a fraction of the eukaryotic diversity, most model organisms including budding yeast and mammals belong to this group.

We began our analysis by performing sequence comparisons of Sct1 and Gpt2 proteins, which represent the entire GPAT complement in *Saccharomyces cerevisiae* [11], [21]. We surveyed 43 complete databases from publicly available genome sequencing projects representing a wide diversity of both fungal and metazoan lineages, and included major branching points in the opisthokont clade (Figure 2). Our survey retrieved high-scoring hits for Sct1/Gpt2 in all fungal species sampled, with the exception being *E. cuniculi*, a parasite with a reduced and compact genome [38]. Surprisingly, Sct1/Gpt2 homologues were also identified in nonfungal unicellular opisthokonts from the Ichthyosporea, Filasterea and Choanozoa (Figure 2). The pattern of conservation that emerged from our analysis excluded all descendant metazoan lineages and displayed a restricted distribution encompassing fungi, basal opisthokonts as well as *T. trahens*, a representative of the Apusozoa thus serving as an outgroup to the analysis.

**Figure 3:**
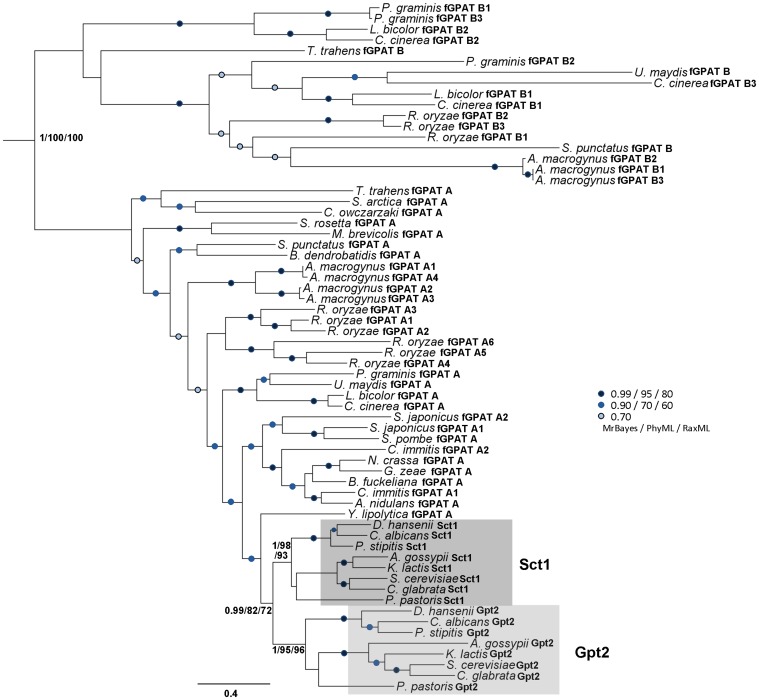
Phylogeny of fungal GPAT homologues found in opisthokonts. Phylogenetic tree of the fungal GPATs. In this and all other phylogenies, node support values are shown in order of Bayesian posterior probabilities, PhyML bootstrap percentages and RaxML bootstrap percentages, or are symbolized as dots, colorized as per the inset to indicate statistical strength. Clades for orthologues of Sct1 and Gpt2 are shaded. For protein accession numbers see Table S3.

Phylogenetic analyses provided further insight into the evolutionary relationships of the putative yeast-like GPATs retrieved by our homology searches. Our study produced two distinct deep-branching clades strongly supported by all three phylogenetic analysis methods used (Figure 3). These two clades each contained a putative fungal GPAT (fGPAT) from the outgroup *T trahens* taxon and clearly separated the closest yeast homologues (fGPAT-A proteins) from a second fGPAT related group (fGPAT-B proteins) only detected in members of Basidiomycota and fungal basal opisthokonts. The largest clade displayed the resolved branching order of all fGPAT-A and Sct1/Gpt2 homologues. Surprisingly, the duplication giving rise to the Sct1/Gpt2 paralogues was restricted to the Saccharomycetaceae lineages that diverged after the split with *Yarrowia*. Altogether these results are indicative of the presence of yeast-like GPATs in deep rooted lineages of Opisthokonta, which were conserved and diversified in the descendant fungal lineages but sculpted by gene loss in the metazoan ones.

### Searching for human mitochondrial GPATs identify a novel metabolic enzyme in fungi

A complex picture has emerged from mammals where two known GPAT systems coexist, one localized to the ER (GPAT3 and 4) and the other associated with mitochondria (GPAT1 and 2) [10]. In addition, a peroxisomal DHAPAT is also present in animals [12]. In contrast, the yeast GPAT enzymes Sct1 and Gpt2 are both ER residents [16], although Gpt2 has also been identified as part of the lipid droplet (LD) proteome under stationary growth conditions [39]. Moreover, GPAT activity has been detected in yeast mitochondria [40], but to date no mitochondrial GPAT has been identified in this organism. In fact, no orthologues of any metazoan GPAT have been described so far in fungi. We have exhausted homology searches in *S. cerevisiae* looking for a putative mitochondrial GPAT, but were curious to investigate if such an enzyme could be found in other fungi.

**Figure 4:**
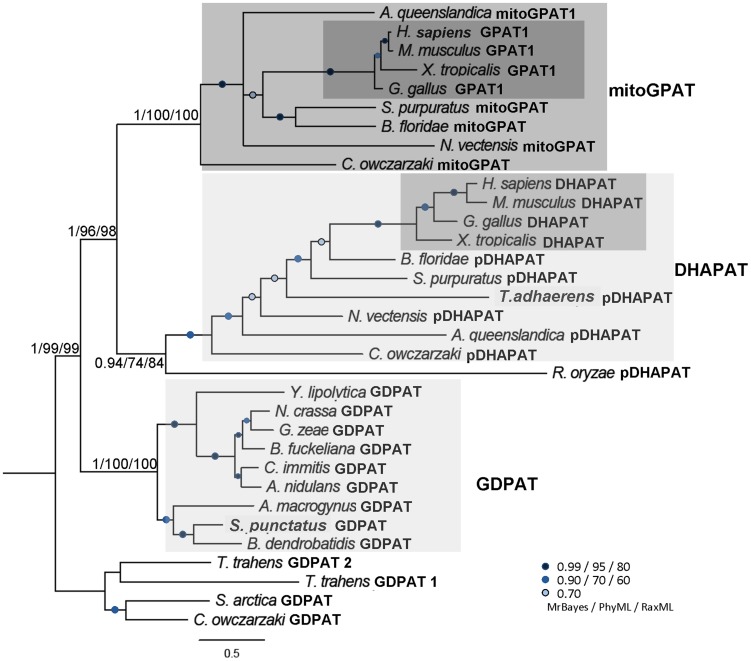
Phylogenetic tree of GDPAT-related genes in opisthokonts. The clades of mitochondrial GPATs (called mitoGPAT, then enumerated), putative DHAPATs (called pDHAPAT, then enumerated) and fungal GDPATs are shaded. Darker shading in each cluster highlights biochemically characterized enzymes from vertebrates, which includes mammalian GPAT1 and DHAPAT homologues. For protein accession numbers see Table S4.

Indeed, a comparative genomic analysis within the opisthokonts using human mitochondrial GPAT1 as a query retrieved a large number of hits in Ascomycota as well as in basal lineages on both fungal and metazoan sides. It is important to note that these proteins were missing from *S. cerevisiae* and the rest of the members previously referred to as the “SACK” (*S. cerevisiae, Ashbya gossypii, Candida glabrata, Kluyveromyces lactis*) clade [41] as well as in all Basidiomycota surveyed (Figure 2). Unexpectedly, the best scoring sequence in reciprocal BLAST searches of fungal hits was the human DHAPAT. To further investigate this relationship, all predicted GPAT and DHAPAT protein sequences identified were subjected to phylogenetic analysis. Since GPAT2 is a divergent form of the mitochondrial enzymes that appeared exclusively in chordate lineages (Figure S1), it was excluded from subsequent analyses. A tree of GPAT/DHAPAT homologues clearly showed a robust grouping of DHAPATs in a clade separated from mitochondrial GPATs, supporting their common origin and a divide of the two branches within the Holozoa, prior to the divergence of *Capsaspora* from the Metazoa (Figure 4). Interestingly, a well defined fungal cluster that preceded the appearance of the duplicated metazoan branches emerged from this analysis. These fungal genes, together with the homologues in the basal outgroups, are designated as GDPATs (G3-P or DHAP acyltransferases) to denote their pre-duplicate status to the DHAPAT and mitoGPAT clades and to highlight our lack of prediction of substrate preference. Upon inspection, a likely peroxisomal target sequence type I (PTS I) was detected in the carboxy-end of the putative novel fungal GDPATs (Figure 2B). A summary of the peptides and strength of the predicted targeting signal by PTS1 Predictor software [42] is presented in Table S2. Our findings strongly suggest the presence of a novel pathway for glycerolipid synthesis in fungal lineages, ancestral to those currently linked to peroxisomes and mitochondria in metazoans.

### Searching for human ER-associated GPATs identify a vertebrate-specific duplication and basal fungal orthologues

**Figure 5:**
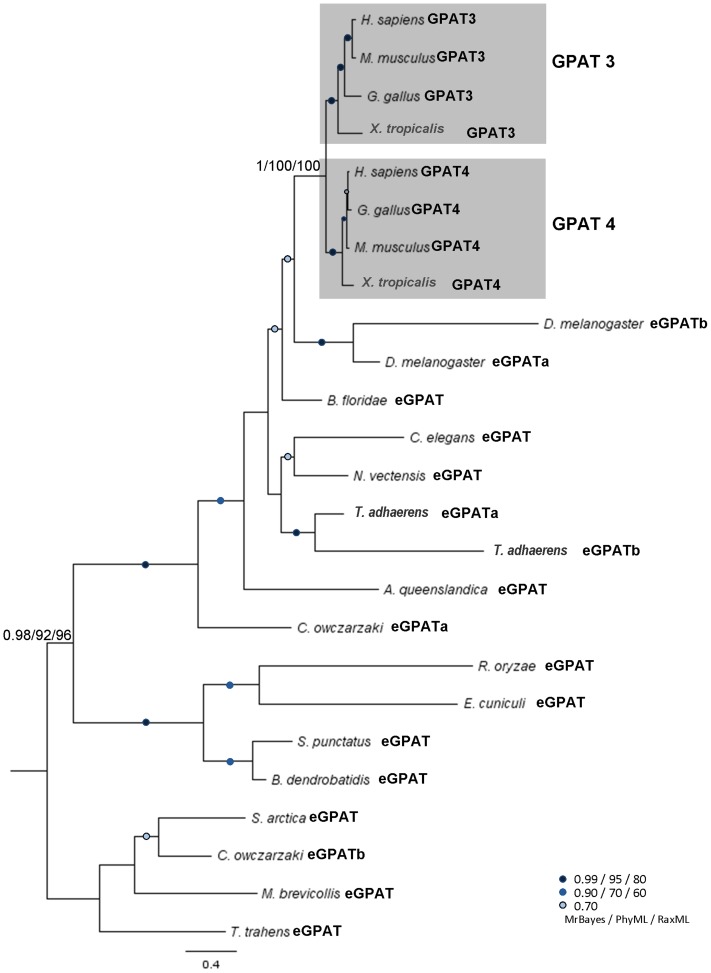
Phylogenetic tree of mammalian eGPATs in opisthokonts. Phylogenetic tree of the emergence of mammalian GPAT3 and GPAT4 homologues (called eGPATs, then enumerated). For protein accession numbers see Table S5.

The last set of known human GPATs so far lacking orthologues in budding yeast are the microsomal GPATs, GPAT3 and GPAT4. Therefore we decided to examine their distribution within the opisthokonts, exploring the possibility that these enzymes may also be present in fungi. This analysis retrieved at least one high-scoring hit for the human microsomal enzymes GPAT3 or GPAT4 in all metazoan lineages (Figure 2). In a pattern similar to that observed for yeast microsomal GPATs, homologues of the human enzymes were also detected in basal lineages of Opisthokonta. This comprised not only all basal lineages on the metazoan side but also all basal lineages on the fungal side, including Microsporidia (*E. cuniculi*). Furthermore, the presence of this GPAT form was also discovered in the outgroup *T. trahens* genome. Homology searching confirmed the absence of these proteins in Basidiomycota and Ascomycota samples. Phylogenetic analyses were then performed in order to describe the relationships between these putative microsomal GPATs and those from mammals. A well-resolved phylogeny was obtained (Figure 5), showing clustering of GPAT3 exclusively with their counterpart GPAT4 sequences (1.00/100/100) indicative of a split within the chordate lineage, between *X. laevis* and *D. melanogaster* sampling points. It is worth noting that the pair of proteins from *D. melanogaster* previously designated as GPAT3 and GPAT4 [43] were excluded from these branches and in fact preceded the appearance of the vertebrate split. These are not directly orthologous to the chordate GPAT 3 and 4 paralogues, and consequently they should not be considered equivalent to GPAT3 or GPAT4 in the literature. We have therefore renamed them eGPATs (eGPATa & eGPATb) in this work.

### Comparison of signature motifs between characterized and novel acyltransferases identified in this study

The discovery of new putative GPATs evolutionarily related to either yeast or mammalian-like enzymes provided us with novel protein sequences from distant organisms to search for unique signature features of each GPAT subclasses. We therefore sought to identify the four diagnostic motifs critical to their enzymatic function [18], [20] and hoped they may provide insight into residues potentially important to substrate specificity and catalysis within each acyltransferase subclass. We were especially interested in the possibility of gaining some insight into biochemical features of the novel group of fungal GDPATs, which represent preduplicates of the metazoan DHAPATs and mitochondrial GPATs.

**Figure 6:**
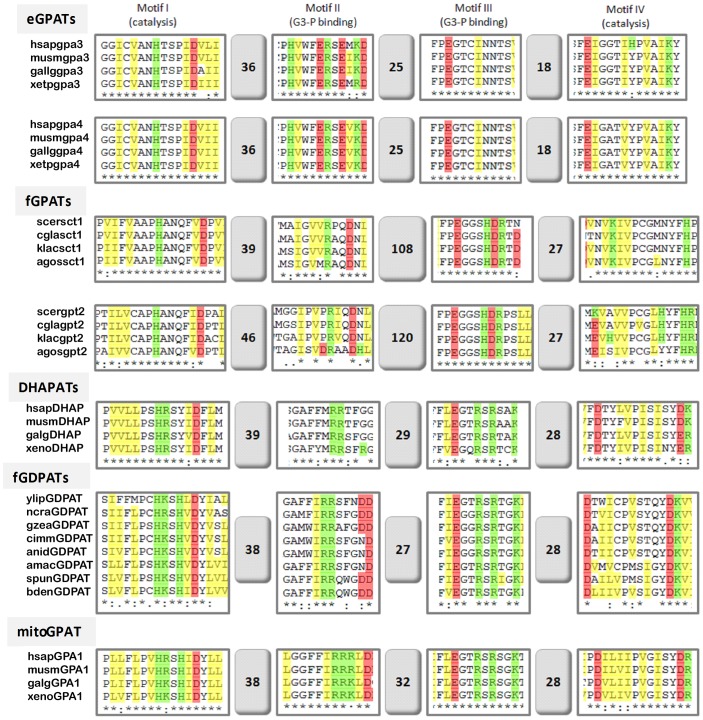
Sequence alignments of the catalytic motifs in strongly supported orthologs of biochemically characterized acyltransferases in each of the eGPATs, fungal GPATs (fGPATs-SACK), mitoGPATs and DHAPATs groups were compare to those of fungal GDPATs. The numbers in the boxes represent the distance between motifs in number of amino-acid residues. Proteins were aligned using Uniprot Align software and acyltransferase motifs recognized as proposed in [18]. Positive, negative and aliphatic residues are highlighted in green, red and yellow respectively. Abbreviations: agos, *Ashbya gossypii*; amac, *Allomyces macrogynus*; anid, *Aspergillus nidulans*; bden, *Batrachochytrium dendrobatidis*; cgla, *Candida glabrata*; cimm, *Coccidioidies immitis*; gall, *Gallus gallus*; gzea, *Gibberella zeae*; hsap, *Homo sapiens*; klac, *Kluyveromyces lactis*; musm, *Mus musculus*; ncra, *Neurospora crassa*; scer, *Saccharomyces cerevisiae*; spun, *Spizellomyces punctatus*; xeno or xet, *Xenopus tropicalis*; ylip, *Yarrowia lipolytica*.

Protein sequences from each set of acyltransferases were aligned, the motifs identified and, conserved residues compared across all subclasses (Table 1, Figure 6, Figure S2 and Figure S3). Examination of the consensus sequences revealed some subclass-specific differences (Table 1). Notably, the invariant histidine and aspartate residues in motif I of fungal GPATs (fGPAT-A and B) were consistently separated by five residues (HX_5_D) versus only four (HX_4_D) in all the other acyltransferases analyzed, including the novel fungal GDPATs. Fungal GPAT-As constituted the largest group analyzed (41 sequences, Table 1 and Figure S2) and despite being taxonomically diverse, this set displayed the highest degree of conservation among motifs. Motif I was characterized by unique conservation of QF residues within the HX_5_D sequence, and motif III clearly emerged as the most conserved one with an invariant FPEGGSHD sequence present in all proteins from this cluster (Figure S3). The second largest set of proteins analyzed was the one corresponding to the metazoan eGPATs (25 sequences, Table 1 and Figure S2). Motifs I to IV in this cluster displayed unique features, but one of the most striking differences between fGPAT-As and eGPATs was the conservation of the distance between motifs (DBM). The DBM between motifs II and III was around three times longer in the fGPAT-As (99–132 versus 23–27 residues in fGPAT-As and eGPATs respectively). In addition 27 residues separated motifs III and IV in fGPAT-As, while only 18 residues distanced these motifs in eGPATs (Table 1 and Figure 6). The fact that these distances are highly conserved in GPATs from basal opisthokonts and Apusozoa suggests they may be critical for unique GPAT topological features, which may regulate catalysis, substrate specificity and/or accessibility of substrates given their subcellular localizations and membrane lipid environment.

**Table 1:** Acyltransferase motifs and distance between motifs (DBM) in microsomal yeast-like (fGPAT-A), metazoan ER-like (erGPAT), mitoGPATs, DHAPAT and fungal GDPAT (fGDPAT) sets.

	Motif I (catalysis)^a^	DBM I–II	Motif II (G3-P/DHAP binding)^a^	DBM II–III	Motif III (G3-P/DHAP binding)^a^	DBM III–IV	Motif IV (catalysis- acyl-CoA binding?)^a^
**fGPAT-As (41)**	**H** x N Q F φ **D**	**35–46**	φ π V x **R** [π/φ] xD	**97–132**	**F** P **E G** G S H D	**26–28**	φ φ **P**[V/C] G
**fGPAT-Bs (9)**	**H** x N x φ φ **D**	**36–40**	φ P φ x **R** x xx	**21–104**	**F** P **E G** [T/G/I] S [H/Y] x	**29–35**	φ φ **P**[V/C/T] π
**eGPATs (25)**	**H** [T/S] [S/T] [P/φ] φ **D**	**36–42**	φ [W/M] F [-/N] **R** x	**23–27**	**F** P **E** **G** T C φ N N	**18**	φ x **P** φ A
**mitoGPATs (10)**	**H** [+] S H φ **D**	**38–39**	F F I [+] **R** [+] φ	**30–32** *	**F** φ **E G** [G/T/S] R [S/T] R	**28**	φ φ **P** φ [π/N]
**fungal GDPATs (9)**	**H** [+] S H φ **D**	**38**	φ φ I R **R** x φ	**27**	**F** φ **E** **G** [G/T] R [S/T] R	**28**	φ x **P** φ π
**DHAPATs (12)**	**H** [+] [S/T] Y φ **D**	**38–39**	F φ M R **R** [T/S] F	**28–30**	**F** φ **E** **G** T R S R	**28**	φ x **P** φ π

All proteins identified in this work for each category were aligned using Uniprot Align software and acyltransferase motifs recognized as proposed in [18]. Number of proteins containing all four motifs used for each alignment is shown in brackets.

a as proposed in [18].

Nomenclature used as defined in [54]:

π  =  P, G, A and S (short chain).

φ  =  Hydrophobic amino acids (V, I, L, F, W, Y and M).

Residues highlighted in red are the hallmark of each motif.

* *N.vectensis* mGPAT [XP_001640722.1] and *C. owczarzaki* mGPAT gi|320164443| have 65 residues between Motifs 2&3.

Consistent with their evolutionary relationship, the motifs in mitoGPATs (10 sequences), fungal GDPATs (9 sequences) and DHAPATs (12 sequences) were very similar across all three subclasses but clearly differentiated from the microsomal eGPATs and fGPATs. Subtle differences in conserved residues in motifs I and II suggest the catalytic features of the novel fungal GDPATs are closer to those of the mitochondrial GPATs than to the peroxisomal DHAPATs (Table 1), but this needs to be assessed by biochemical characterization of members of this cluster.

## Discussion

Our molecular evolutionary analysis has identified the repertoire of enzymes catalyzing the first biosynthetic step of all glycerolipids in organisms corresponding to key branching points in the evolution of Fungi and Metazoa. This offers significant insights into the unique features and roles that these enzymes may play in the maintenance of lipid homeostasis, particularly in eukaryotes with important implications for the rational targeting of lipid pathways for biotechnological and medical manipulation of lipid content and lipid species. Our work should also have important implications in the area of synthetic biology where current attempts towards a synthetic full reconstruction of a minimal cell rely exclusively on the glycerolipid synthesizing capacity of the system. Efforts have been directed towards inducing the self-reproduction of the external membrane by solely expressing a GPAT and the second acyltransferase involved in phosphatidic acid synthesis [44], [45]. Understanding the contribution of different isoforms during evolution should improve the outcome of these attempts.

### Reductive evolution and sculpting of GPATs in opisthokonts

A surprising finding from our investigations is the realization that eukaryotic ancestors of opisthokonts likely displayed a more complex array of GPATs compared to some of their descendants. This can be interpreted as a model of reductive evolution followed by sculpting via gene loss in fungi and metazoan lineages. We have identified four ancient groups of acyltransferases present in lineages named i) fungal GPAT-A, ii) fungal-GPAT-B) iii) mammalian ER-like GPATs (eGPATs), and iv) GDPATs, which then duplicated sometime in the holozoa into iv-a) mitochondrial GPATs (mitoGPATs) and iv-b) DHAPATs (Figure 2). Interestingly, nearly the whole suite of acyltransferases was represented in the amoeboid symbiont *Capsaspora owczarzaki* (Figure 2) a filasterean that occupies a pivotal phylogenetic position between the closest relatives of Metazoa (choanoflagellate protists), and Fungi [46]. In fact, both “fungal-specific” and “metazoan-specific” microsomal GPATs were found in all basal opisthokont lineages analyzed in this work as well as in the Apusozoa member used as the outgroup, *T. trahens*. Indeed, the situation could well be even more complicated, as in the phylogenies for both the ‘metazoan’ mitochondrial/peroxisomal and microsomal homologues, there appeared to be an extra set of paralogues in the basal holozoan lineages that grouped with the apusozoan outgroup. These could be explained as deep paralogues, retained in these lineages, but lost in fungi and Metazoa/choanoflagellates independently. However, they could also be divergent homologues that have been mis-placed due to phylogenetic artifact. Rather than invoking further complicated scenarios, we will acknowledge that these will need to be accounted for, preferably once better taxon sampling of this region of the eukaryotic tree is available.

Although this early complexity and evolutionary maintenance of redundancy remains unclear, it has been proposed that genetic robustness related to metabolic networks, reflects the ability of an organism to survive in different environments [47]. The richness of acyltransferases found in basal holozoan and holomycetozoan taxa is exciting and may reveal a degree of metabolic versatility and adaptation to changing environments, as well as lipid biochemistry, yet to be understood.

**Figure 7:**
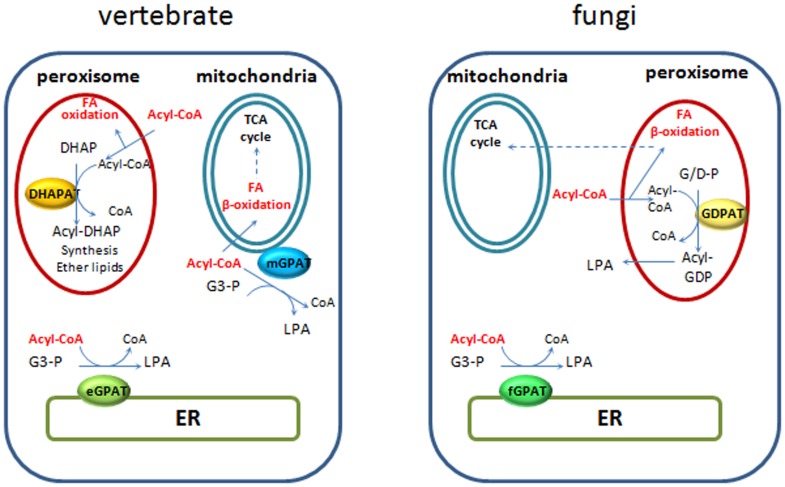
Intracellular distribution of phosphatidic acid biosynthetic and fatty acid oxidation pathways in vertebrate and fungi.

The maintenance of a pre-existing type of microsomal acyltransferase and loss of the other type during metazoan and fungi evolution probably reflects key roles of these proteins in cellular processes unique to each of these lineages. While the microsomal GPATs displayed restricted distributions, the larger family of mitoGPATs, putative DHAPATs and novel GDPATs spread among all metazoan and several fungi lineages analyzed. This represents a novel finding of our study, as no such proteins were previously described in fungi, potentially indicative of an undescribed metabolic capacity in this lineage. Our phylogenetic analysis clearly established a close relationship between peroxisomal DHAPATs and mitochondrial GPATs which may be reflective of the functional and evolutionary interactions between these organelles [48]. It is worth noting the cluster of mitochondrial related GPATs was confined to basal and metazoan lineages and was not represented in fungi. Co-existence with DHAPATs was observed in all organisms displaying at least one mitochondrial related isoform (Figure 2). It is well known peroxisomal DHAPATs are involved in the synthesis of ether glycerolipids in mammals, but these lipids have not been described so far in fungi, suggesting the putative peroxisomal GDPATs play a different role in fungi. Furthermore, close analysis of the catalytic fungal GDPATs motifs I and II (Table 1 and Figure 6) shows that these enzymes share more similarity to mitoGPATs than to DHAPATs at these positions. Biochemical characterization of members of this group will be necessary to unambiguously determine their substrate specificity. It has been previously hypothesized that mitochondrial GPATs control the availability of fatty acids for mitochondrial β-oxidation in mammals [26], [49]. In most fungi, fatty acid β-oxidation occurs in peroxisomes or hybrid pathways between peroxisomes and mitochondria [50], therefore it is tempting to speculate diversion of FAs from this pathway could be a role played by these novel fungal GDPATs predicted to be localized to peroxisomes in organisms relying heavily on fatty acid metabolism (Figure 7). In line with this rationale, we found two organisms with only one acyltransferase, *E. cuniculi*, an obligate intracellular parasite and the free-living fission yeast *S. pombe* which colonizes sugar-rich environmental niches. In both cases a predicted microsomal GPAT supplied the essential function; a microsomal enzyme in *E. cuniculi* or a yeast-like one in *S. pombe*. Furthermore, data available from PomBase [51] indicates that deletion of this GPAT in fission yeast produces a lethal phenotype (ref. # SPBC1718.04), confirming no other enzyme is able to compensate for the loss of its function. Interestingly, these two organisms lack pathways for FA β-oxidation [50] which could represent a contributing factor to the selection of a unique microsomal GPAT during evolution, as the acyltransferase would not need to compete for acyl-CoA substrates at different subcellular locations. In other words, because these organisms have no beta oxidation, there is no need to regulate it, hence the lack of GDPATs or mitoGPATs in these cases. It is worth noting that in mammals mitochondrial GPATs are localized to the outer mitochondrial membrane and may compete with the carnitine acyl transferase system for the short, medium and long acyl-CoA pools. In contrast, peroxisomal DHAPATs are localized inside the organelle and may restrict peroxisomal fatty acid oxidation to very long chain fatty acid pools (Figure 7).

### Redundancy in modern GPATs

Our comparative genomic and phylogenetic analysis has shed some light into the complex evolutionary dynamics related to the origin of budding yeast and mammalian GPATs, which are currently the focus of many biochemical, biological and physiological studies. The split into the yeast GPAT genes *SCT1* and *GPT2* was found to be confined to Saccharomycotina (Figure 2 and 3) and was the result of a small-scale duplication. This kind of duplication has been proposed to support a greater likelihood of neofunctionalization compared to whole genome duplications [52]. In a similar trend, evolutionary innovation was also observed in vertebrates with splits in mitochondrial GPAT1 and GPAT2 as well as microsomal GPAT3 and GPAT4 (Figure 5 and Figure S1). Unique contributions of each isoform to specific cellular processes and physiology have been described ([19], [27], [43], [53]) but progress in our understanding of protein structure, molecular determinants of substrate specificity and enzymatic mechanism has been limited due to the challenge posed by the membrane bound nature of these proteins and lack of success in obtaining protein crystals.

Nonetheless, specific protein structural features emerged from the comparison of sequence alignments within our expanded repertoire of GPATs and DHAPATs. In particular, the conservation of the distance between catalytic motifs in enzymes, from organisms separated by hundreds of millions of years of evolution as in the case between basal opisthokonts and animals or fungi (Table 1), was striking. These conserved distances probably reflect important topological features that impact the spatial relationship between the acyl-CoA and glycerol-3-phosphate in the GPAT active sites but their functional significance should be proven experimentally and further investigated.

## Conclusions

Overall our comparative genomic and phylogenetic analyses were able to address major evolutionary questions about GPAT enzymes in opisthokonts. Specifically, we report that the *S. cerevisiae* and human GPAT complements are not entirely representative of the fungal and holozoan complements respectively. Particularly the discovery of a GDPAT-related clade of fungal genes with putative peroxisomal targeting, potentially represents an undescribed metabolic faculty in this lineage. The overall distribution of GPAT homologues is suggestive of reductive evolution, with high complexity in the ancestors of the opisthokont clade, followed by loss and sculpting of the complement in the descendent lineages. Since the vast majority of characterization of these central metabolic enzymes in eukaryotes has taken place in the yeast and mammalian systems, this study allows for work in each of these well-established model systems to be better applied both to the other and to the members of their respective lineages. It also reveals, that gene loss, as well as expansion, has taken place in sculpting the biosynthetic lipid repertoire in the lineages giving rise to our best studied and best known model eukaryotes.

## Supporting Information

Figure S1 **Phylogenetic tree of mitochondrial GPATs.** The emergence of mitochondrial GPAT1 and GPAT2 in vertebrates is shaded.
(TIF)

Figure S2 **Sequence alignments of the catalytic motifs in strongly supported orthologs of eGPATs.** Proteins were aligned using Uniprot Align software and acyltransferase motifs recognized as proposed in [18]. Positive, negative and aliphatic residues are highlighted in green, red and yellow respectively. Abbreviations: egp, erGPAT; ampq, *Amphimedon queenslandica*; bdet, *Batrachochytrium dendrobatidis*; bfld, *Branchiostoma floridae*; capo, *Capsaspora owczarzaki*; celg, *Caenorhabditis elegans*; dmel, *Drosophila melanogaster*; enzn, *Encephalitozoon cuniculi*; gall, *Gallus gallus*; hsap, *Homo sapiens*; mbre, *Monosiga brevicolis*; musm, *Mus musculus*; nemv, *Nematostella vectensis*; rhiz, *Rhizopus oryzae*; sarc, *Sphaeroforma árctica*; spiz, *Spizellomyces punctatus*; tadh, *Trichoplax adhaerens*; ttra, *Thecamonas trahens*; xetp, *Xenopus tropicalis*.
(TIF)

Figure S3 **Sequence alignments of the catalytic motifs in strongly supported orthologs of fungal GPATs.** Proteins were aligned using Uniprot Align software and acyltransferase motifs recognized as proposed in [18]. Positive, negative and aliphatic residues are highlighted in green, red and yellow respectively. Abbreviations: agos, *Ashbya gossypii*; ama, *Allomyces macrogynus*; anid, *Aspergillus nidulans*; bdet, *Batrachochytrium dendrobatidis*; bfuc, *Botryotinia fuckeliana*; cal, *Candida albicans*; cgla, *Candida glabrata*; cimm, *Coccidioidies immitis*; ccin, *Coprinopsis cinérea*; cowc, *Capsaspora owczarzaki*; dha, *Debaryomyces hansenii*; enzn, *Encephalitozoon cuniculi*; gza, *Gibberella zeae*; klac, *Kluyveromyces lactis*; lbi, *Laccaria bicolor*; mbre, *Monosiga brevicolis*; ncra, *Neurospora crassa*; pipa, *Pichia pastoris*; pis, *Pichia stipitis*; puc or pgra, *Puccinia graminis*; rhiz, *Rhizopus oryzae*; sarc, *Sphaeroforma arctica*; scer, *Saccharomyces cerevisiae*; sja, *Schizosaccharomyces japonicus*; spom, *Schizosaccharomyces pombe*; spun, *Spizellomyces punctatus*; sros, *Salpingoeca rosetta*; tadh, *Trichoplax adhaerens*; ttra, *Thecamonas trahens*; umay, *Ustilago maydis*; ylip, *Yarrowia lipolytica*.
(TIF)

Table S1 **List of species and specific strains used in the proteomic analysis of opisthokont GPATs.**
(DOCX)

Table S2 **PTS1 predictions.**
(XLSX)

Table S3 **fGPATs-accession numbers.**
(XLSX)

Table S4 **GDPATs-DHAPATs and mitoGPATs- accession numbers.**
(XLSX)

Table S5 **eGPATs- accession numbers.**
(XLSX)
