# Supplementary figures and images for: Phylogenetic Analysis of Glycerol 3-Phosphate Acyltransferases in Opisthokonts Reveals Unexpected Ancestral Complexity and Novel Modern Biosynthetic Components
Source: PLoS One. 2014 Oct 23;9(10):e110684. doi: 10.1371/journal.pone.0110684 (PMC4207751; doi:10.1371/journal.pone.0110684)

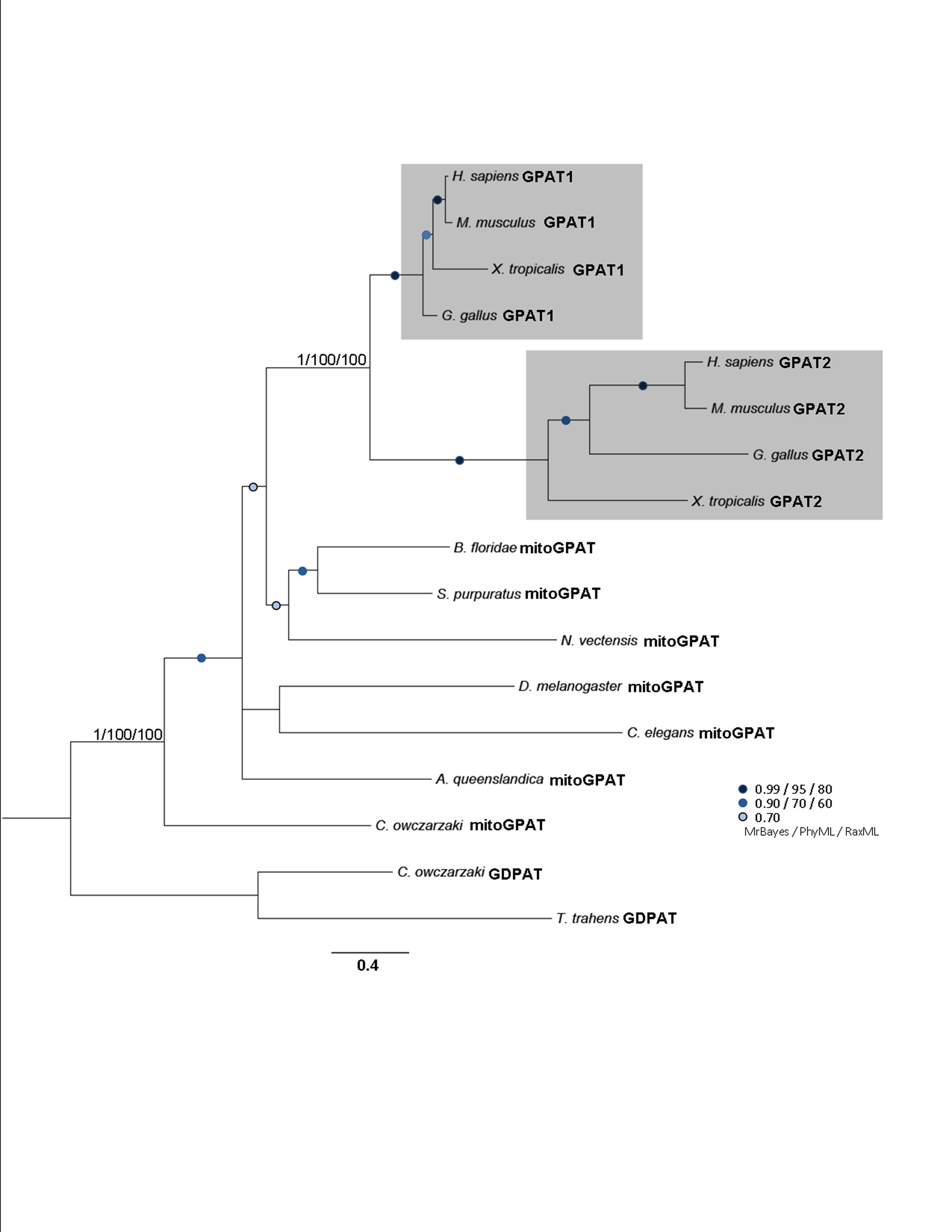

Supplement: Figure S1 — Phylogenetic tree of mitochondrial GPATs. The emergence of mitochondrial GPAT1 and GPAT2 in vertebrates is shaded. (TIF) [file pone.0110684.s001.tif]

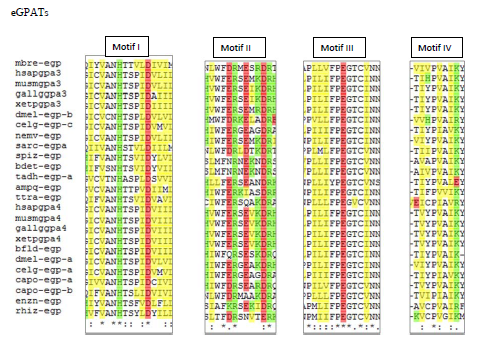

Supplement: Figure S2 — Sequence alignments of the catalytic motifs in strongly supported orthologs of eGPATs. Proteins were aligned using Uniprot Align software and acyltransferase motifs recognized as proposed in [18]. Positive, negative and aliphatic residues are highlighted in green, red and yellow respectively. Abbreviations: egp, erGPAT; ampq, Amphimedon queenslandica; bdet, Batrachochytrium dendrobatidis; bfld, Branchiostoma floridae; capo, Capsaspora owczarzaki; celg, Caenorhabditis elegans; dmel, Drosophila melanogaster; enzn, Encephalitozoon cuniculi; gall, Gallus gallus; hsap, Homo sapiens; mbre, Monosiga brevicolis; musm, Mus musculus; nemv, Nematostella vectensis; rhiz, Rhizopus oryzae; sarc, Sphaeroforma árctica; spiz, Spizellomyces punctatus; tadh, Trichoplax adhaerens; ttra, Thecamonas trahens; xetp, Xenopus tropicalis. (TIF) [file pone.0110684.s002.tif]

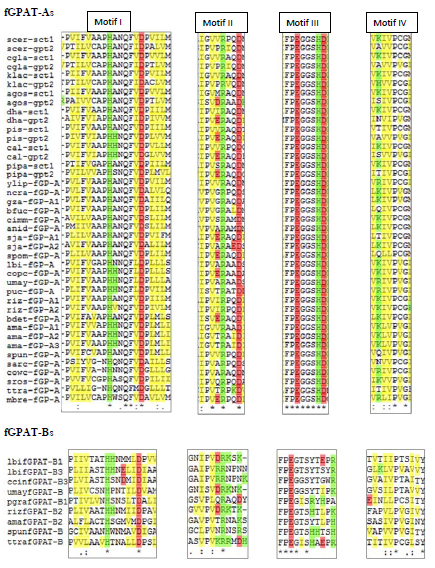

Supplement: Figure S3 — Sequence alignments of the catalytic motifs in strongly supported orthologs of fungal GPATs. Proteins were aligned using Uniprot Align software and acyltransferase motifs recognized as proposed in [18]. Positive, negative and aliphatic residues are highlighted in green, red and yellow respectively. Abbreviations: agos, Ashbya gossypii; ama, Allomyces macrogynus; anid, Aspergillus nidulans; bdet, Batrachochytrium dendrobatidis; bfuc, Botryotinia fuckeliana; cal, Candida albicans; cgla, Candida glabrata; cimm, Coccidioidies immitis; ccin, Coprinopsis cinérea; cowc, Capsaspora owczarzaki; dha, Debaryomyces hansenii; enzn, Encephalitozoon cuniculi; gza, Gibberella zeae; klac, Kluyveromyces lactis; lbi, Laccaria bicolor; mbre, Monosiga brevicolis; ncra, Neurospora crassa; pipa, Pichia pastoris; pis, Pichia stipitis; puc or pgra, Puccinia graminis; rhiz, Rhizopus oryzae; sarc, Sphaeroforma arctica; scer, Saccharomyces cerevisiae; sja, Schizosaccharomyces japonicus; spom, Schizosaccharomyces pombe; spun, Spizellomyces punctatus; sros, Salpingoeca rosetta; tadh, Trichoplax adhaerens; ttra, Thecamonas trahens; umay, Ustilago maydis; ylip, Yarrowia lipolytica. (TIF) [file pone.0110684.s003.tif]
