# Supplementary material for: Phylogenetic Analysis of Glycerol 3-Phosphate Acyltransferases in Opisthokonts Reveals Unexpected Ancestral Complexity and Novel Modern Biosynthetic Components
Source: PLoS One. 2014 Oct 23;9(10):e110684. doi: 10.1371/journal.pone.0110684 (PMC4207751; doi:10.1371/journal.pone.0110684)
Supplement: Table S1 — List of species and specific strains used in the proteomic analysis of opisthokont GPATs. (DOCX) [file pone.0110684.s004.docx]

**Table S1** Names of species and specific strains used in the proteomic analysis of opisthokont GPATs. Whole reference sequence protein databases were downloaded from the NCBI [1] or The Broad Institute [2] in FASTA format.

| Species | Strain | Taxid | Source |
| --- | --- | --- | --- |
| *Allomyces macrogynus* | Allomyces macrogynus ATCC 38327 | 578462 | Broad Institute |
| *Amphimedon queenslandica* | Amphimedon queenslandica | 400682 | NCBI |
| *Ashbya gossypii* | Ashbya gossypii FDAG1 | 1034331 | NCBI |
| *Aspergillus nidulans* | Aspergillus nidulans FGSC A4 | 227321 | NCBI |
| *Batrachochytrium dendrobatidis* | Batrachochytrium dendrobatidis JAM81 | 684364 | NCBI |
| *Botryotinia fuckeliana* | Botryotinia fuckeliana B05.10 | 332648 | NCBI |
| *Branchiostoma floridae* | Branchiostoma floridae | 7739 | NCBI |
| *Caenorhabditis elegans* | Caenorhabditis elegans | 6239 | NCBI |
| *Candida albicans* | Candida albicans SC5314 | 237561 | NCBI |
| *Candida glabrata* | Candida glabrata CBS 138 | 284593 | NCBI |
| *Capsaspora owczarzaki* | Capsaspora owczarzaki ATCC 30864 | 595528 | NCBI |
| *Coccidioidies immitis* | Coccidioides immitis RS | 246410 | NCBI |
| *Coprinopsis cinerea* | Coprinopsis cinerea okayama7#130 | 240176 | NCBI |
| *Debaryomyces hansenii* | Debaryomyces hansenii CBS767 | 284592 | NCBI |
| *Drosophila melanogaster* | Drosophila melanogaster | 7227 | NCBI |
| *Encephalitozoon cuniculi* | Encephalitozoon cuniculi GB-M1 | 284813 | NCBI |
| *Gallus gallus* | Gallus gallus | 9031 | NCBI |
| *Gibberella zeae* | Gibberella zeae PH-1 | 229533 | NCBI |
| *Homo sapiens* | Homo sapiens | 9606 | NCBI |
| *Kluyveromyces lactis* | Kluyveromyces lactis NRRL Y-1140 | 284590 | NCBI |
| *Laccaria bicolor* | Laccaria bicolor S238N-H82 | 486041 | NCBI |
| *Monosiga brevicolis* | Monosiga brevicolis | 431895 | NCBI |
| *Mus musculus* | Mus musculus | 10090 | NCBI |
| *Nematostella vectensis* | Nematostella vectensis | 45351 | NCBI |
| *Neurospora crassa* | Neurospora crassa OR74A | 367110 | NCBI |
| *Pichia pastoris* | Komagataella pastoris CBS 7435 | 981350 | NCBI |
| *Pichia stipitis* | Scheffersomyces stipitis CBS 6054 | 322104 | NCBI |
| *Puccinia graminis* | Puccinia graminis f. sp. tritici CRL 75-36-700-3 | 418459 | NCBI |
| *Rhizopus oryzae* | Rhizopus oryzae RA 99-880 | 246409 | NCBI |
| *Salpingoeca rosetta* | Salpingoeca sp. ATCC 50818 | 946362 | NCBI |
| *Schizosaccharomyces japonicus* | Schizosaccharomyces japonicus yFS275 | 402676 | NCBI |
| *Schizosaccharomyces pombe* | Schizosaccharomyces pombe 972h- | 284812 | NCBI |
| *Sphaeroforma arctica* | Sphaeroforma arctica JP610 | 667725 | Broad Institute |
| *Spizellomyces punctatus* | Spizellomyces punctatus DAOM BR117 | 645134 | Broad Institute |
| *Strongylocentrotus purpuratus* | Strongylocentrotus purpuratus | 7668 | NCBI |
| *Thecamonas trahens* | Thecamonas trahens ATCC 50062 | 461836 | Broad Institute |
| *Trichoplax adhaerens* | Trichoplax adhaerens | 10228 | NCBI |
| *Ustilago maydis* | Ustilago maydis 521 | 237631 | NCBI |
| *Xenopus tropicalis* | Xenopus (Silurana) tropicalis | 8364 | NCBI |
| *Yarrowia lipolytica* | Yarrowia lipolytica CLIB122 | 284591 | NCBI |

**References**

1. Benson DA, Karsch-Mizrachi I, Lipman DJ, Ostell J, Sayers EW (2009) GenBank. Nucleic Acids Res 37: D26–31. doi:10.1093/nar/gkn723.

2. Ruiz-Trillo I, Burger G, Holland PWH, King N, Lang BF, et al. (2007) The origins of multicellularity: a multi-taxon genome initiative. Trends Genet 23: 113–118. doi:10.1016/j.tig.2007.01.005.
